# Identification of biomarkers associated with CD8+ T cells in rheumatoid arthritis and their pan-cancer analysis

**DOI:** 10.3389/fimmu.2022.1044909

**Published:** 2022-11-24

**Authors:** Zhenyu Zhao, Jie Ren, Siping Xie, Longchun Zou, Qianyue Zhao, Shan Zeng, Dingsheng Zha

**Affiliations:** ^1^ Department of Orthopedics, The First Affiliated Hospital, Jinan University, Guangzhou, China; ^2^ Department of Rheumatology, The First Affiliated Hospital, Jinan University, Guangzhou, China; ^3^ Department of Medical Records, The First Affiliated Hospital, Jinan University, Guangzhou, China; ^4^ School of Stomatology, Jinan University, Guangzhou, China; ^5^ School of Basic Medicine and Public Health, Jinan University, Guangzhou, China

**Keywords:** CD8+ T Cells_1_, rheumatoid arthritis_2_, WGCNA_3_, Pan-Cancer_4_, ssGSEA_5_

## Abstract

**Introduction:**

Rheumatoid arthritis (RA), a prevailing chronic progressive autoimmune disease, seriously affects the patient's quality of life. However, there is still a lack of precise treatment and management methods in clinical practice. Previous studies showed that CD8+ T cells take a lead in the progression of RA.

**Methods:**

Genes closely related to CD8+T cells in RA were identified through multiple RA datasets, CIBERSORT, and WGCNA algorithms. Further machine learning analysis were performed to identify CD8+T cell-related genes most closely related to RA. In addition, the relationship between these three key genes and 33 cancer species was also explored in this study.

**Results:**

In this study, 10 genes were identified to be closely related to CD8+T cells in RA. Machine learning analysis identified 3 CD8+T cell-related genes most closely related to RA: CD8A, GZMA, and PRF1.

**Discussion:**

Our research aims to provide new ideas for the clinical treatment of RA.

## Introduction

Rheumatoid arthritis (RA) is a common chronic autoimmune disease associated with systemic inflammatory processes. This chronic damage first affects the patient’s bones and joints (1). The progression of the disease will affect the patient’s quality of life, bringing a huge economic burden to individuals and society. Another prominent manifestation of RA is systemic inflammatory lesions outside the joints, in particular, the digestive system, nervous system, cardiovascular system, etc (2). Furthermore, because of the significant variability of RA disease, it is challenging to distinguish it clinically from other autoimmune disorders (such as systemic lupus erythematosus, Sjögren’s syndrome, etc.) (3) and face challenges in the administration of chemotherapy (4). In the developed world, RA affects between 0.5 and 1% of adults, or about 24.5 million people, with 5 to 50 new cases per 100,000 people every year (5, 6). The aforementioned issues require immediate attention given the significant prevalence of RA in the general population.

CD8+ T cells, also known as cytotoxic T cells, play an important role in the elimination of malignant cells and intracellular inflammation in the body (7). Current research shows that in the face of infection, inflammation, tumor, and other pathological conditions, the metabolic level of CD8+ T cells and the anti-inflammatory phenotype exhibited by the body will undergo various changes (8). Some studies show that its epigenetic modifications also alter (9). The current consensus is that CD8+ T cells promote the progression of RA by releasing pro-inflammatory and cytolytic mediators (10, 11). The study by M Margarida Souto-Carneiro et al. (12) mentions that metabolic demands in hypoxic tissues sustain the continued damage of this cell to the joint. A study by Helena Carvalheiro et al. (10) revealed the full landscape of CD8+ T cells in RA, revealing that CD8+ T cells are characterized by upregulation and secretion of inflammatory mediators throughout RA. The above studies all illustrate the important role of CD8+ T cells in the progression of RA and may become an excellent intervention target for clinical RA treatment.

In this study, based on the results of the CIBERSORT algorithm and weighted gene correlation networks analysis (WGCNA) analysis, we finally identified 10 key CD8+ T cell-related genes. The three genes most closely related to CD8+ T cell infiltration in RA, namely CD8A, GZMA, and PRF1, were found to be associated with a variety of cancers. This points out a new path for the treatment of RA.

## Material and methods

### Collection and processing of gene expression data

The GSE55235 (13), GSE1919 (14), GSE48780 (15), GSE55457 (13), and GSE55584 (13) datasets were retrieved and downloaded from the Gene Expression Omnibus(GEO, https://www.ncbi.nlm.nih.gov/geo/). Limma package was used to normalize the data and convert between probe ID and gene symbol through platform information, removing probes without gene symbol and taking the average expression value of multiple probes under the same symbol (16). We used variation coefficients to select the most significantly varied genes for analysis.

### Analysis of immune cell infiltration

The proportion of 22 immune cell types and the infiltration of immune cells in samples from GSE55235, GSE1919, GSE48780, GSE55457, and GSE55584 datasets was calculated using the CIBERSORT algorithm (17).

### Construction of WGCNA co-expression network

“WGCNA” was used to construct a gene co-expression network for genes in the GSE55235 dataset with a variation coefficient greater than 0.1. The top 25% of highly expressed variants were analyzed (18). The reliability of the constructed scale-free network is ensured by removing abnormal samples. For that, they were used to approximate appropriate soft threshold rates and obtain adjacency values between genes whose variances were more significant before applying power functions. The adjacency values were then converted into topological overlap matrices (TOM) and derived the dissimilarity (1-TOM) values. The dynamic tree-cutting method was finally used to identify modules by hierarchical clustering of genes.

### Building blocks feature relation

Each module underwent component analysis according to its features, and the correlation between module characteristics and T-cell subtypes was then analyzed using the Pearson test. We considered the module significantly correlated with T cell subtypes when P<0.05. The central module was defined as that with the highest correlation coefficient with CD8+T cells.

### Hub gene selection and validation

Based on the module connectivity and clinical characteristic relationships of each gene in the central module, candidate central genes were chosen. Pearson’s correlation between genes’ absolute values is used to describe module connectivity. The overall Pearson’s correlation between each gene and each characteristic was used to identify the relationship between clinical traits. For candidate central genes, we set gene significance >0.75. Verify whether there is a significant correlation between the candidate hub genes and CD8+T, to determine the reliable hub genes. GSE1919, GSE48780, GSE55457, and GSE55584 datasets were used to verify the Spearman correlation between CD8+T cell and central gene expression. Hub genes were defined as candidate hub genes that were significantly associated with CD8+T cells in the GSE55235, GSE1919, GSE48780, GSE55457, and GSE55584. In addition, CIBERSORT, MCPCOUNTER, QUANTISEQ, TIMER, and XCELL algorithms were used to verify the correlation between genes and CD8+T cells in different tumors.

### Identification of key genes using LASSO regression and random forest

Critical genes for T cell CD8+ were identified using the LASSO regression and random forest algorithms. Least Absolute Shrinkage and Selection Operator (LASSO) regression is performed using the “glmnet” package. RF is implemented using the “randomForest” package (19). A Venn diagram was used to visualize the results of the two algorithms and obtain the intersection genes.

### Functional and pathway enrichment analysis

We examined genes using Gene Ontology (GO) analysis (20), which comprised molecular function (MF), cellular component (CC), and biological process (BP), and Kyoto Encyclopedia of Genes and Genomes (KEGG) pathway analysis (21).

### Hub gene correlation

Genes GSE55235, GSE1919, GSE48780, GSE55457, and GSE55584 were correlated, and the analysis results were then visualized using “ggplots2”. We obtained high-throughput expression data of cancer and normal tissues from the TCGA (22) and GETx databases (http://commonfund.nih.gov/GTEx), respectively, and evaluated the correlation between genes in various tumors and various normal tissues to further illustrate the correlation between central genes.

### The association between genes and transcription factors

Transcription factors are involved in various complex biological processes by regulating the transcription process through specific DNA sequence recognition. RNA-seq data were obtained from TCGA database and we used ChEA3 (https://maayanlab.cloud/chea3/) database to identify the transcription factors of hub gene and demonstrated them using Cytoscape software.

### Gene set enrichment analysis

A gene set’s significance between two biological states can be determined using gene set enrichment analysis (GSEA) (23). A single gene GSEA analysis was carried out to better investigate the potential mechanism through which Hub genes affect RA. At the same time, h.all.v7.5.1.symbols.gmt in the Molecular Signatures Database (MSigDB: https://www.gsea-msigdb.org/gsea/index.jsp) was selected as the reference gene set, and Spearman rank correlation coefficient was obtained by using the package “corrplot”, and adjustment P value <0.05 was used as the screening criterion.

### Construction of protein-protein interaction network

GeneMANIA (http://genemania.org/), a website for creating protein-protein interaction (PPI) networks, may be used to make predictions for the functions of genes and find genes with similar effects. Among them, providing physical interaction, co-expression, co-localization, gene enrichment analysis, genetic interaction, and locus prediction are some of the bioinformatics methods used by network integration algorithms.

## Results

### RA gene expression data acquisition and evaluation of immune cells infiltration

Microarray expression data of rheumatoid arthritis (GSE55235) were downloaded from the GEO database, and genes with a variation coefficient greater than 0.1 were selected for subsequent analysis. We next used the R package “CIBERSORT” to analyze the corresponding expression data in the dataset GSE55235 to ascertain the percentage of various immune cell subtypes in each sample from the dataset. The proportion of each block’s seven T cell subtypes was then chosen as the trait data for the WGCNA.

### Construction of WGCNA co-expression network

**Figure 1:**
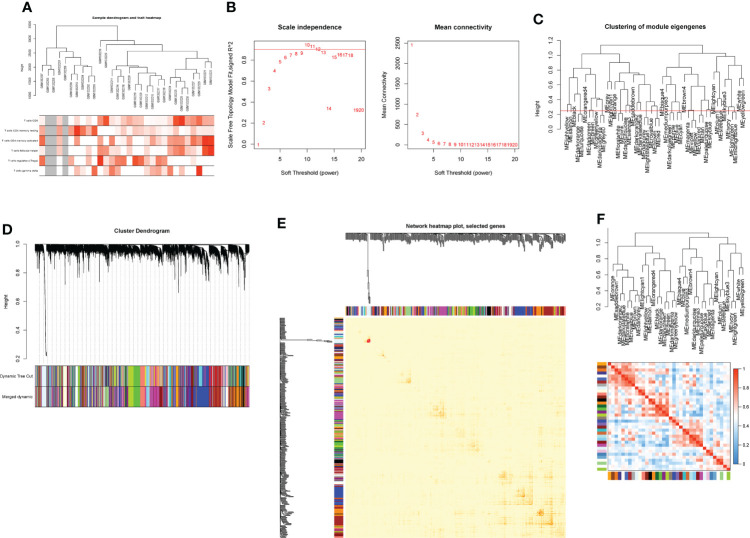
Construction of WGCNA co-expression network. **(A)** Sample clustering dendrogram with tree leaves corresponding to individual samples. **(B)** Soft threshold β = 7 and scale-free topological fit index (R2). **(C)** Clustered dendrograms were cut at a height of 0.25 to detect and combine similar modules. **(D)** Shows the original and combined modules under the clustering tree. **(E)** Collinear heat map of module feature genes. Red color indicates a high correlation, blue color indicates the opposite results. **(F)** Clustering dendrogram of module feature genes.

**Figure 2:**
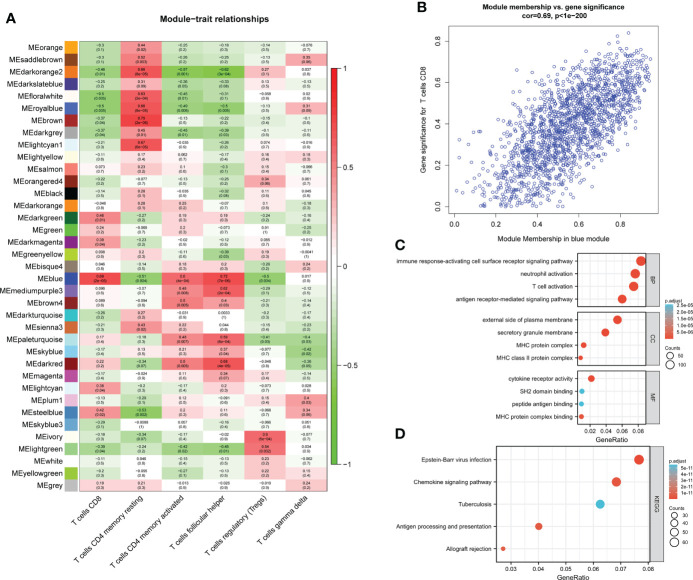
module–trait correlations and Functional enrichment analysis **(A)** Heat map of module–trait correlations. Red represents positive correlations and blue represents negative correlations. **(B)** Module Membership in the blue module. **(C)** GO analysis. **(D)** KEGG analysis.

Based on genes with a variation coefficient greater than 0.1 from the GSE55235 dataset, the R package “WGCNA” was used to construct a gene co-expression network. The average association coefficient and Pearson correlation coefficient were then calculated, and cluster analysis on all samples in the dataset was performed. A scale-first network was built using β=7 as the soft threshold rate (**Figures 1A, B**). To create hierarchical clustering trees, the dynamic hybrid cutting technique was employed. Each branch is a module that combines all genes with similar expression levels, and each leaf represents a gene (**Figures 1C, D**). Functionally equivalent modules were then combined into one large module, resulting in 37 modules (**Figures 1E, F**, **2A**).

### Hub module identification and function enrichment analysis

Among the 37 modules, the blue module had the most significant relationship with CD8+T cell (R^2 =^ 0.69, P=2E-05). We designate the blue module as the hub module as a result. In this module, potentially important genes that are most closely related to CD8+ T cells were shown to have a stronger correlation. Gene with gene significance >0.75 were selected as candidate central genes, and a total of 10 genes (NKG7, CD8A, DHRS9, CCL5, IL2RG, TNS3, PARP12, GZMA, PRF1, CYTH4) were selected (**Figure 2B**).

The genes in the blue module were then subjected to GO/KEGG analysis. The findings demonstrated that genes for immune response-activating cell surface receptor signaling pathway, antigen receptor-mediated signaling pathway, and T cell activation in BP were highly enriched in the blue module. The external side of the plasma membrane, MHC protein complex, and secretory membrane were significantly enriched in CC. While cytokine receptor activity, MHC protein complex binding, and SH2 domain binding were significantly enriched in MF (**Figure 2C**). Module genes were significantly enriched in Epstein-Barr virus infection, Chemokine signaling pathway, Antigen processing, and presentation in KEGG (**Figure 2D**).

### Screening and identification of hub genes

**Figure 3:**
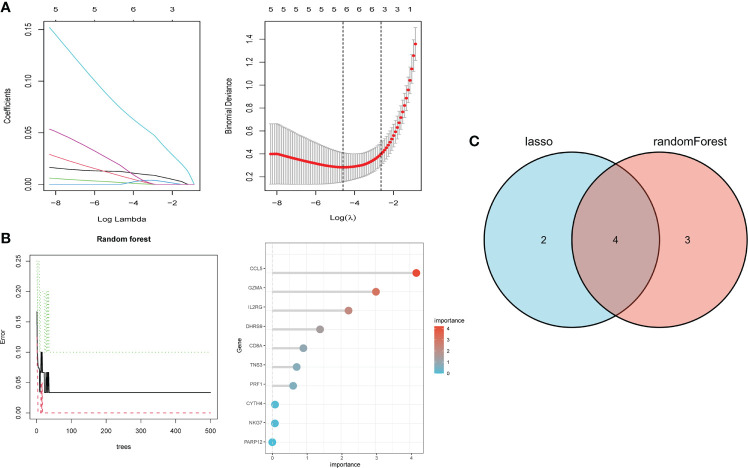
Hub gene selection. **(A)** Adjustment of feature selection in the minimum absolute shrinkage and selection operator model (lasso). **(B)** randomForest error rate versus the number of classification trees, the top 20 relatively important genes. **(C)** Three algorithmic Venn diagram screening genes.

**Figure 4:**
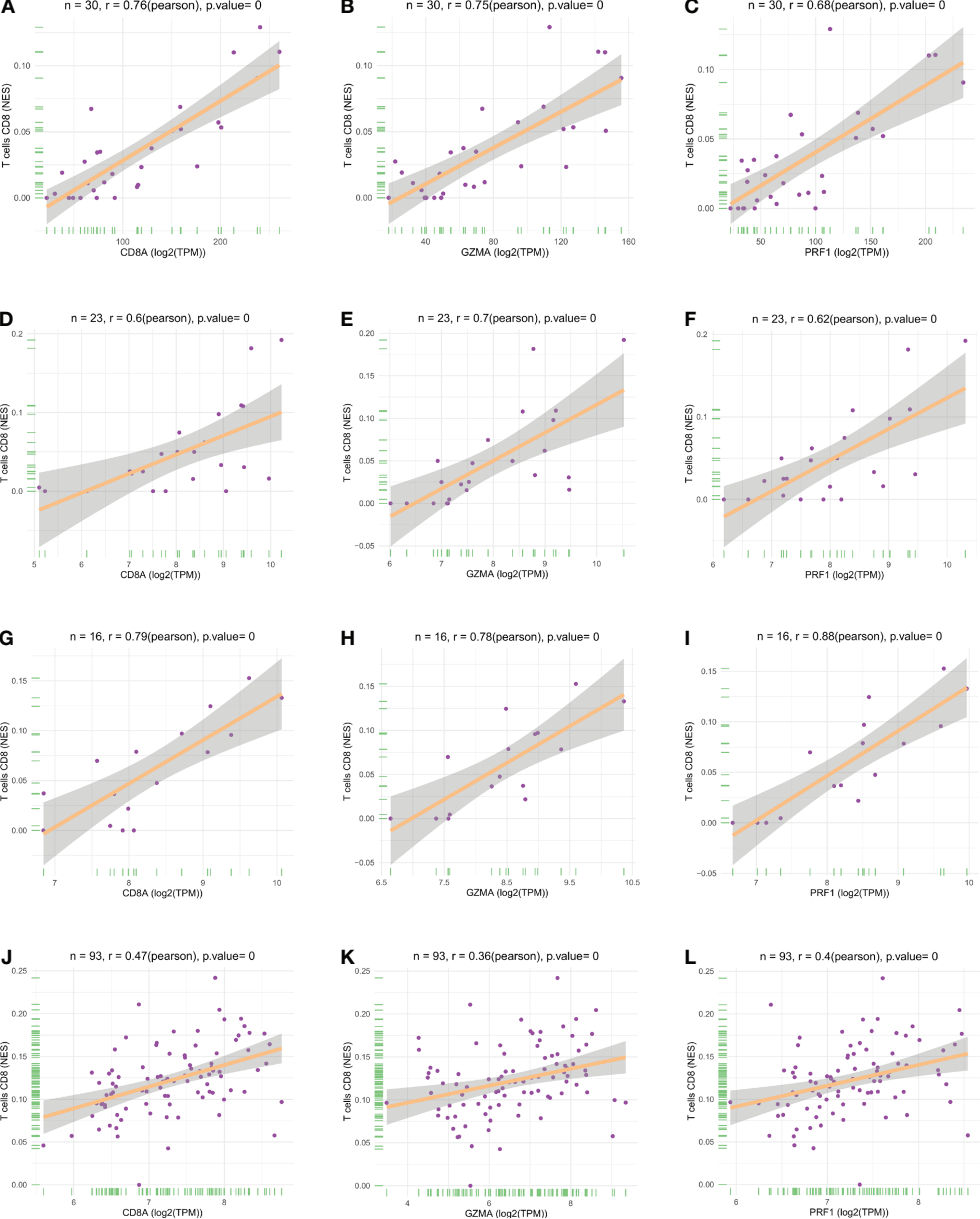
Scatter plots of hub gene expression versus CD8+ T cell infiltration levels in three different datasets: **(A-C)** GSE55235. **(D-F)** GSE55457. **(G-I)** GSE55584. **(J-L)** GSE1919 and GSE48780.

**Figure 5:**
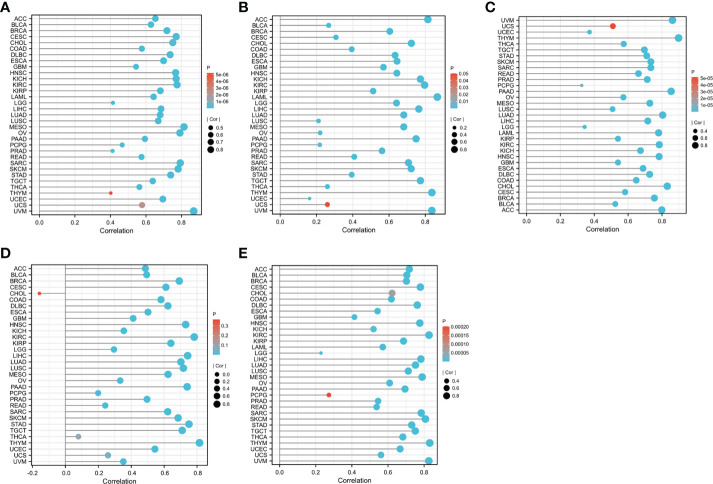
The relationship between CD8A expression levels and the degree of CD8+ T cell infiltration in different cancers was statistically significant at P<0.05: **(A)** CIBERSORT. **(B)** MCP-counter. **(C)** QUANTISEQ. **(D)** TIMER. **(E)** XCELL.

We performed LASSO and random forest analysis on the above-mentioned 10 genes, and a total of 6 genes (NKG7, CD8A, TNS3, PARP12, GZMA, PRF1) were obtained by LASSO analysis (**Figure 3A**). Genes of Importance >0.6 were selected from RF analysis results for analysis (CD8A, DHRS9, CCL5, IL2RG, TNS3, GZMA, PRF1)(**Figure 3B**). Next, a Venn diagram was used to visualize the intersection genes of LASSO and RF, and a total of 4 genes (CD8A, TNS3, GZMA, PRF1) were found (**Figure 3C**). GSE55235 (**Figures 4A–C**), GSE55457 (**Figures 4D–F**), GSE55584 (**Figures 4G–I**), GSE1919 and GSE48780 (**Figures 4J–L**) were used to identify and validate the correlation between the levels of CD8A, TNS3, GZMA, PRF1, and CD8+ T-cell infiltration. CD8A, GZMA, and PRF1 were identified as reliable Hub genes. The results of the analysis revealed that three genes were significantly positively correlated with the degree of CD8+ T-cell infiltration in these data sets. At the same time, we selected different cancers for analysis (**Figure 4**). CIBERSORT, McP-counter, QUANTISEQ, TIMER, and XCELL were employed to explore the expression values of genes related to CD8+T cells in different cancers and utilized the “ggPlot” R package for visualization. It was discovered that CD8A (**Figure 5**), GZMA (**Supplementary Figure 1**), and PRF1 (**Supplementary Figure 2**) were positively correlated with CD8+T cell infiltration in various malignancies. These analyses verified that the identified hub genes play a significant role in the tumor immune microenvironment and are highly correlated with the degree of CD8+ T-cell infiltration.

### Hub gene correlation

**Figure 6:**
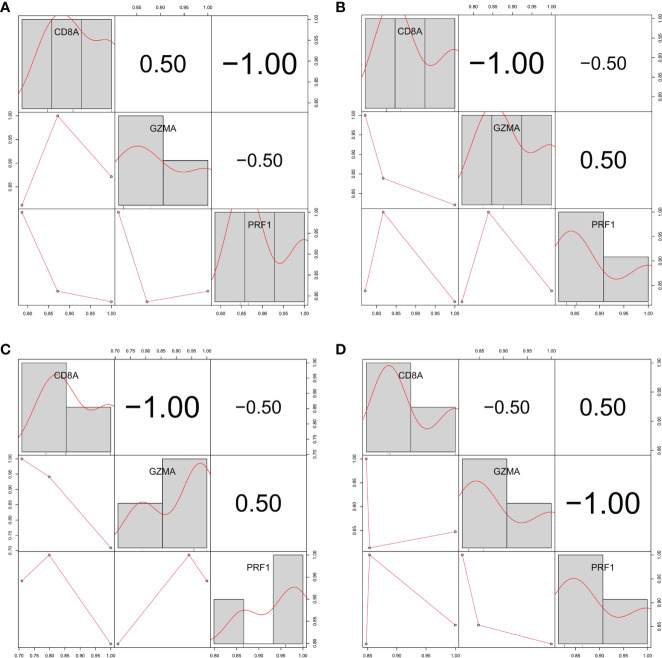
Relationship between the expression levels of three hub genes in three different gene sets **(A)** GSE55235. **(B)** GSE55457. **(C)** GSE55584. **(D)** GSE1919 and GSE48780.

**Figure 7:**
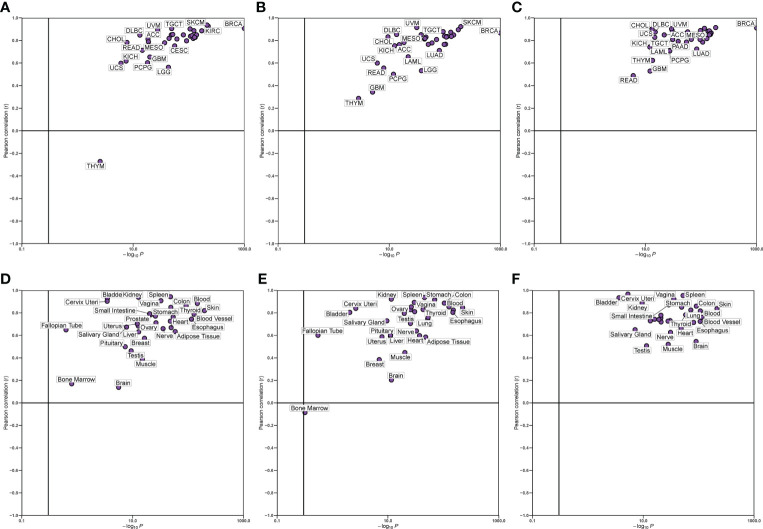
Relationship between the expression levels of three hub genes. **(A-C)** In different cancer types. **(D-F)** In normal tissues.

We verified the correlation between hub genes in different data sets and found that CD8A, GZMA, and PRF1 were significantly positively correlated with each other. The relationship between CD8A, GZMA, and PRF1 in various malignancies was then examined (**Figure 6**). Except for THYM, CD8A, and GZMA being positively correlated in all tumors, CD8A and PRF1 were positively correlated in 33 tumors, and GZMA and PRF1 were positively correlated in 33 tumors (**Figures 7A–C**). The results of normal tissue analysis showed that CD8A and GZMA were positively correlated in various normal tissues, GZMA and PRF1 were positively correlated in various normal tissues, CD8A and PRF1 were negatively correlated in Bone Marrow, and the rest were positively correlated (**Figures 7D–F**).

### Transcription factor analysis

**Figure 8:**
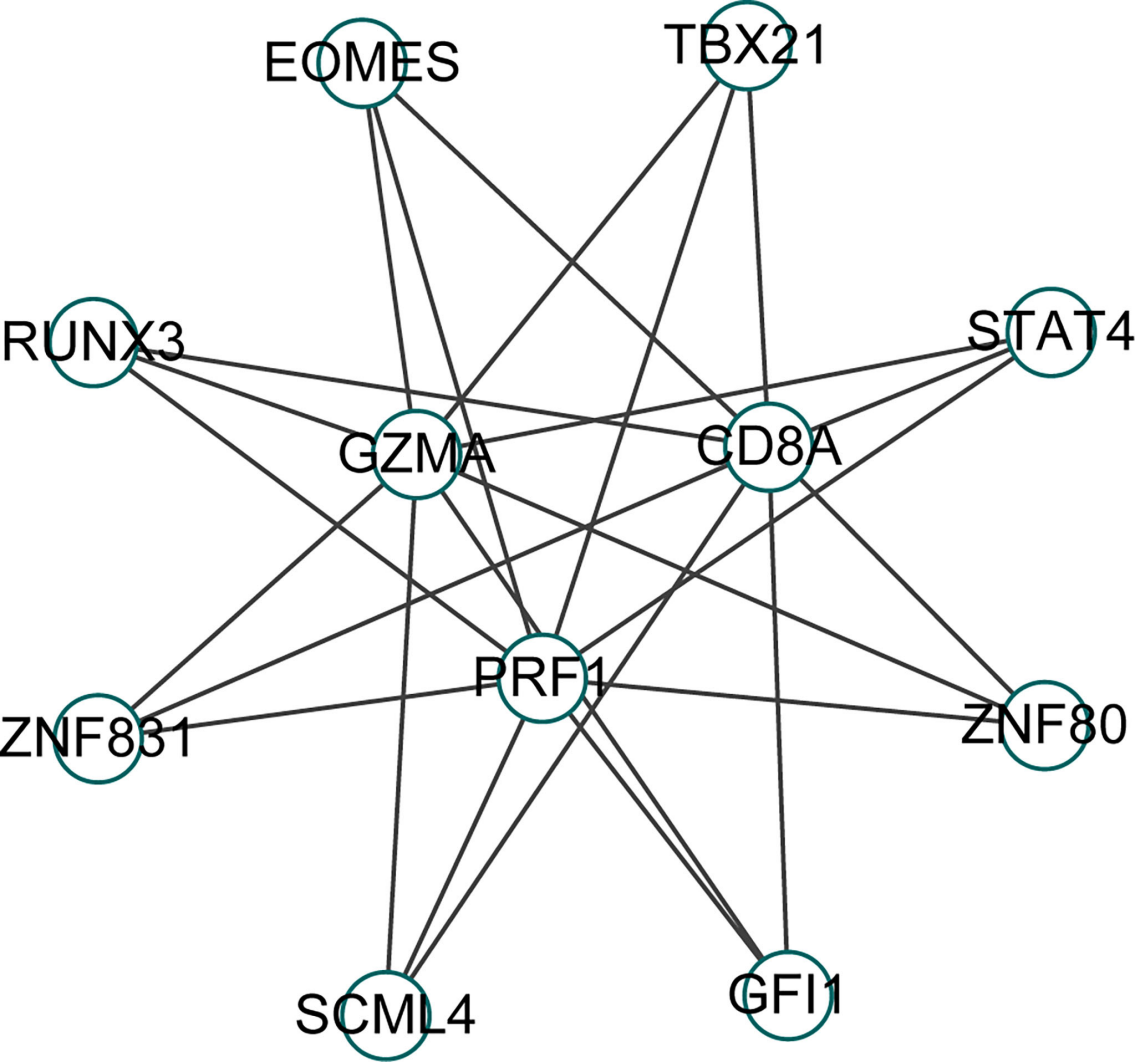
Prediction of transcription factors of Hub gene in CHEA3 database.

We identified 8 common transcription factors (EOMES, TBX21, STAT4, ZNF80, GFI1, SCML4, ZNF831, RUNX3) associated with CD8A, GZMA, and PRF1 from the CHEA3 database (**Figure 8**).

### Gene enrichment

**Figure 9:**
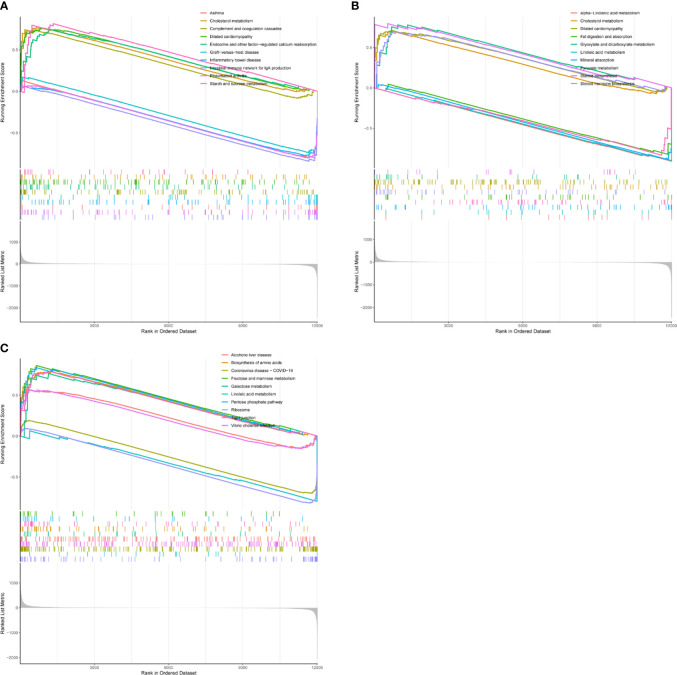
GSEA analysis of hub gene. **(A)** CD8A. **(B)** GZMB. **(C)** PRF1.

To explore the function of the hub gene, we carried out a single gene GSEA analysis. It was found that CD8A was mainly enriched in Rheumatoid arthritis, Asthma, Cholesterol metabolism, etc (**Figure 9A**). GZEA is mainly enriched in cholesterol metabolism, steroid biosynthesis, mineral absorption, etc (**Figure 9B**). PRF1 was mainly enriched in Coronavirus disease COVID-19, the Pentose phosphate pathway, and Linoleic acid metabolism (**Figure 9C**). We then used h.all.v7.5.1.symbols. gmt as a reference gene set to analyze the correlation between genes and genomes. CD8A, GZMA, and PRF1 were found to be positively correlated with interferon gamma response, interferon alpha response, inflammatory response, il6 jak stat3 signaling, complement, and allograft rejection, and negatively with uv response dn, pancreas beta cells, myogensis, cholesterol homeostasis, androgen response, and adipogenesis.

### Analysis of hub gene interaction

**Figure 10:**
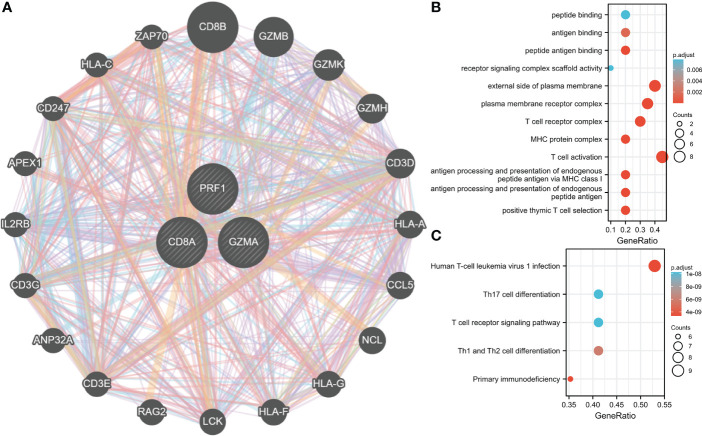
Interaction analysis of Hub genes. **(A)** Characterized gene co-expression network. **(B)** GO analysis of co-expressed genes. **(C)** Co–expressed gene KEGG analysis.

GeneMANIA database was employed to create PPI networks for Hub genes. To further investigate the function of hub genes, we constructed a 20-gene interaction network (**Figure 10A**). At the same time, 20 genes were investigated for functional enrichment. Results showed that, in BP, Genes were mainly enriched in T cell-mediated immunity, modification of morphology, or physiology of other organisms. Genes were mainly enriched in immunological synapses and the external side of the plasma membrane under CC. In MF, genes were mainly enriched in phospholipase activator activity and lipase activator activity (**Figure 10B**). Genes in the KEGG pathway may regulate Primary immunodeficiency, Viral protein interaction with cytokine, and cytokine receptor. These findings lead us to hypothesize those hub genes are crucial for the immune system (**Figure 10C**).

## Discussion

RA is a chronic progressive autoimmune disease with an increasing incidence that cause great damage to patients’ exercise and labor ability. But unfortunately, there are still no effective intervention measures in clinical practice, and there is also a lack of precise diagnosis and treatment methods. A large number of previous studies have shown that CD8+ T cells play an extremely important role in the progression of RA and the clinical outcome of patients (10, 24). Based on this, genes in the GSE55235 dataset were combined with WGCNA and a variety of machinery. The learning algorithm finally obtained three core genes closely related to CD8+ T cells, namely CD8A, GZMA, and PRF1. We further performed validation and analysis on these three genes.

Interestingly, we found that these three core genes were mentioned in previous studies in RA, among which GZMA has the most relevant studies. GZMA is a member of the serine protease family, mainly derived from NK cells and T cells, and plays an important regulatory role in cell death and the release of inflammatory mediators (25). As early as a clinical study in 1999 (26), GZMA was found to be highly expressed in the serum and synovial tissue of RA patients, and in 2017, Llipsy Santiago et al. found in mice that knocking out GZMA can reduce osteoclasts. Active and efficient tissue production of collagen-induced arthritis in mice (a mouse model of RA) (27). More interestingly, the role of GZMA is not limited to RA. Abnormal expression of GZMA has been found in autoimmune diseases such as SLE and Sjögren’s syndrome, which shows that the gene has a very objective intervention value (28, 29). For CD8A, this gene is currently considered to be one of the key genes in the differential diagnosis and prognosis prediction of RA in the bioinformatics analysis conducted by several research teams (30, 31), while the study by Cai-Yue Gao et al. found that knocking out the CD8A gene can promote the damage of salivary glands in Sjögren’s syndrome mice, which may also be an important mechanism for resident CD8+ T cells to induce joint synovial damage in RA (32). PRF1 belongs to the perforin family of genes. The study by Lan Wang et al. showed that PRF1 is important for rheumatoid disease, while the study by Zoya Qaiyum et al. found the abnormal expression of this gene in the gene map of ankylosing spondylitis (33).

In addition, the relationship between these three key genes and 33 cancer species was also explored in this study. We found that the CD8A gene is positively associated with a variety of cancers, a result also confirmed by other research groups. For example, Chirag Krishna et al. found that in clear cell renal cell carcinoma, patients with high CD8A expression had more severe ICB resistance symptoms and tumor-associated macrophage infiltration (34); while Bruno Sangro et al. The CD8A gene is a good prognostic predictor in nivolumab-treated patients with advanced hepatocellular carcinoma (35). Similarly, Zhiwei Zhou et al. showed that GZMA can cleave GSDMB and induce tumor cell pyroptosis in an IFN-γ-dependent manner (36). Moreover, PRF1 has also been shown to play a role in the prognosis and progression of various cancers including breast and colon cancer (37, 38). These studies illustrate the important roles of these three genes in human diseases from another dimension.

Since our research is based on bioinformatics methods, it may be subject to the bias of the analysis results due to the quality of the samples in the database. Therefore, more *in vivo* or *in vitro* experiments are needed to further verify the results.

## Data availability statement

The original contributions presented in the study are included in the article/**Supplementary Material**. Further inquiries can be directed to the corresponding authors.

## Author contributions

ZZ and JR planned the research concept and designed it, made provisions for study material, collected data and analyzed them, and wrote and approved the manuscript. SX searched for data and wrote programming code. LZ and QZ collected pictures and graphs as well as wrote response letters. SZ and DZ collected data and analyzed them, wrote and approved, and helped correct the manuscript. All authors contributed to the article and approved the submitted version.

## Funding

This research is funded by the National Natural Science Foundation of China (Project number: 81901650), Shenzhen Key Laboratory of Musculoskeletal Tissue Reconstruction and Function Restoration and Shenzhen People’s Hospital (Project number: ZDSYS20200811143752005), Guangzhou Science and Technology Project (Grant No. 201904010060, Effect and mechanism of S100A4 on collagen-induced arthritis (CIA) model in mice. National Natural Science Foundation of China (Project number:81401766), the fundamental research funds for the central universities (Project number:21619348), Funding by Science and Technology Projects in Guangzhou (Project number: 2021020200460).

## Conflict of interest

The authors declare that the research was conducted in the absence of any commercial or financial relationships that could be construed as a potential conflict of interest.

## Publisher’s note

All claims expressed in this article are solely those of the authors and do not necessarily represent those of their affiliated organizations, or those of the publisher, the editors and the reviewers. Any product that may be evaluated in this article, or claim that may be made by its manufacturer, is not guaranteed or endorsed by the publisher.
