# Supplementary material for: Identification of biomarkers associated with CD8+ T cells in rheumatoid arthritis and their pan-cancer analysis
Source: Front Immunol. 2022 Nov 24;13:1044909. doi: 10.3389/fimmu.2022.1044909 (PMC9730809; doi:10.3389/fimmu.2022.1044909)
Supplement: Supplementary file 1 [file DataSheet_1.docx]

Supplementary Material

## Supplementary Figures


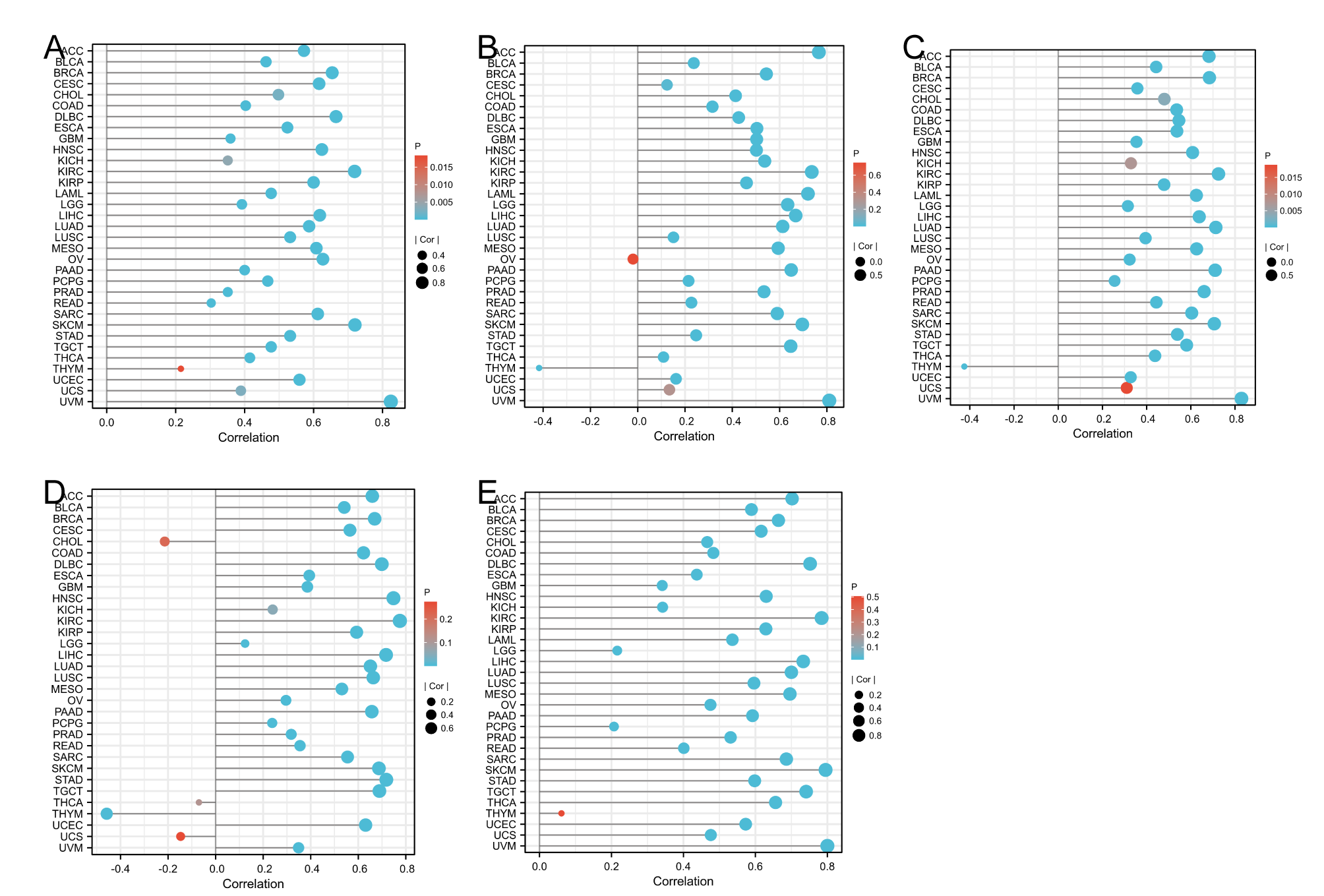


**Supplementary Figure 1.** The relationship between GZMA expression levels and the degree of CD8+ T cell infiltration in different cancers was statistically significant at P<0.05: (A) CIBERSORT. (B) MCP-counter. (C) QUANTISEQ.(D)TIMER.(E)XCELL
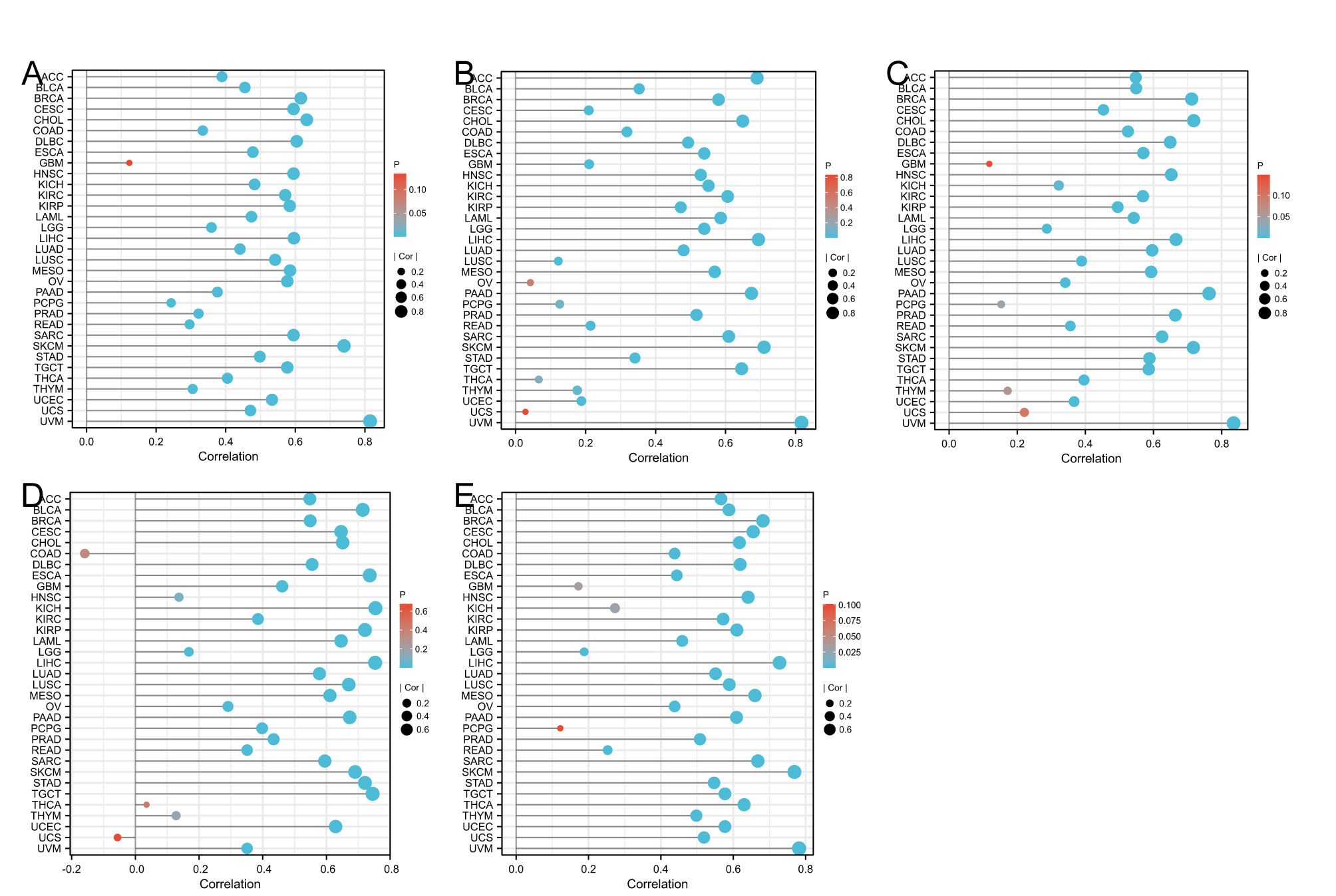


**Supplementary Figure 2.**The relationship between PRF1 expression levels and the degree of CD8+ T cell infiltration in different cancers was statistically significant at P<0.05: (A) CIBERSORT. (B) MCP-counter. (C) QUANTISEQ.(D)TIMER.(E)XCELL
